# β-Ecdysterone Enhanced Bone Regeneration Through the BMP-2/SMAD/RUNX2/Osterix Signaling Pathway

**DOI:** 10.3389/fcell.2022.883228

**Published:** 2022-05-20

**Authors:** Cai-Ping Yan, Xing-Kuan Wang, Ke Jiang, Chong Yin, Chao Xiang, Yong Wang, Chaoyu Pu, Lu Chen, Yu-Ling Li

**Affiliations:** ^1^ Department of Orthopaedics, Affiliated Hospital of North Sichuan Medical College, Nanchong, China; ^2^ Laboratory of Biological Tissue Engineering and Digital Medicine, Affiliated Hospital of North Sichuan Medical College, Nanchong, China; ^3^ Laboratory for Bone Metabolism, Xi’an Key Laboratory of Special Medicine and Health Engineering, Key Lab for Space Biosciences and Biotechnology, NPU-UAB Joint Laboratory for Bone Metabolism, Research Center for Special Medicine and Health Systems Engineering, School of Life Sciences, Northwestern Polytechnical University, Xi’an, China

**Keywords:** β-ecdysterone, bone regeneration, bone morphogenetic protein-2 (BMP2), RUNX 2, Smad

## Abstract

Bone defects are a global public health problem. However, the available methods for inducing bone regeneration are limited. The application of traditional Chinese herbs for bone regeneration has gained popularity in recent years. β-ecdysterone is a plant sterol similar to estrogen, that promotes protein synthesis in cells; however, its function in bone regeneration remains unclear. In this study, we investigated the function of β-ecdysterone on osteoblast differentiation and bone regeneration *in vitro* and *in vivo*. MC3T3-E1 cells were used to test the function of β-ecdysterone on osteoblast differentiation and bone regeneration *in vitro*. The results of the Cell Counting Kit-8 assay suggested that the proliferation of MC3T3-E1 cells was promoted by β-ecdysterone. Furthermore, β-ecdysterone influenced the expression of osteogenesis-related genes, and the bone regeneration capacity of MC3T3-E1 cells was detected by polymerase chain reaction, the alkaline phosphatase (ALP) test, and the alizarin red test. β-ecdysterone could upregulate the expression of osteoblastic-related genes, and promoted ALP activity and the formation of calcium nodules. We also determined that β-ecdysterone increased the mRNA and protein levels of components of the BMP-2/Smad/Runx2/Osterix pathway. DNA sequencing further confirmed these target effects. β-ecdysterone promoted bone formation by enhancing gene expression of the BMP-2/Smad/Runx2/Osterix signaling pathway and by enrichment biological processes. For *in vivo* experiments, a femoral condyle defect model was constructed by drilling a bone defect measuring 3 mm in diameter and 4 mm in depth in the femoral condyle of 8-week-old Sprague Dawley male rats. This model was used to further assess the bone regenerative functions of β-ecdysterone. The results of micro-computed tomography showed that β-ecdysterone could accelerate bone regeneration, exhibiting higher bone volume, bone surface, and bone mineral density at each observation time point. Immunohistochemistry confirmed that the β-ecdysterone also increased the expression of collagen, osteocalcin, and bone morphogenetic protein-2 in the experiment group at 4 and 8 weeks. In conclusion, β-ecdysterone is a new bone regeneration regulator that can stimulate MC3T3-E1 cell proliferation and induce bone regeneration through the BMP-2/Smad/Runx2/Osterix pathway. This newly discovered function of β-ecdysterone has revealed a new direction of osteogenic differentiation and has provided novel therapeutic strategies for treating bone defects.

## Graphical Abstract

Schematic illustrations of the fabrication of the bone defect model and action of β-Ecd in promoting bone regeneration and repair of bone defects. We established a rat model of a femoral bone defect *in vivo* to evaluate the effect of β-Ecd on bone regeneration. Rats injected intraperitoneally with 72 mg/kg β-Ecd showed a higher degree of ossification of regenerated bone tissue at the site of the bone defect at weeks 4 and 8. β-ecdysterone binding to the BMP2 receptor activates SMAD1 to bind to SMAD1/5/8, promotes RUNX2 and OSTERIX replication in the nucleus, and mediates bone regeneration. This study provides a new approach to the treatment of bone injury and degenerative diseases represented by bone defects and osteoporosis.

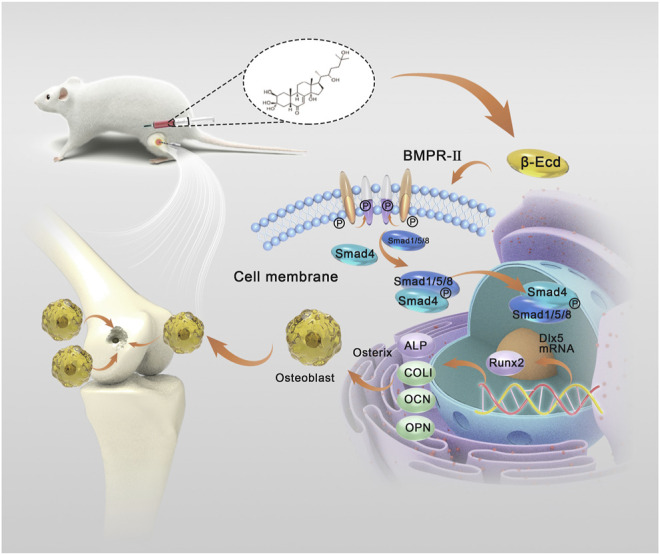

## Introduction

Bone defect refers to the destruction of the structural integrity of the phalanx, and complete or partial destruction of its continuity. Studies have shown that a variety of signal transduction mechanisms regulate bone growth metabolism and regeneration after bone injury. When these critical signal transduction mechanisms that promote bone growth are not fully activated or destroyed, bone formation is reduced and bone marrow fat accumulation increases, resulting in impaired bone regeneration (Hak et al., 2014; Yu et al., 2018). Bone regeneration is a highly complex but organized process that requires damaged bones to return to their pre-injury cellular structure and biomechanical functions (Schindeler et al., 2008). Both, intramembranous and endochondral ossification are essential forms of bone regeneration (Phillips 2005; Takigawa 2013; Ko and Sumner 2021). In the process of intramembranous ossification, bone marrow mesenchymal stem cells (BMSCs) differentiate directly into osteoblasts and deposit mineralized extracellular matrix to achieve bone regeneration (Percival and Richtsmeier 2013). BMSCs are cells with multi-differentiation potential, and have the ability to differentiate into bone, cartilage, fat, nerves, or myoblasts *in vivo* and *in vitro* (Sumer, Liu et al., 2018, Yang et al., 2019; Zhao et al., 2020). BMSCs can also secrete a variety of cytokines (such as BMP-2, IGF-1, IL-6, and M-CSF) to promote bone regeneration (Meirelles et al., 2009). On exposure to certain specific chemical mediators, cytokines, and mechanical stimulation, intracellular BMP-Smad, Wnt/β-catenin, Notch, Hedgehog, or other signaling pathways of BMSCs are activated to promote osteoblast differentiation (Abdallah et al., 2005). However, when the specific environment is destroyed due to various diseases, BMSCs show abnormal osteogenic differentiation, an imbalance of metabolic regulation, which reduces the bone remodeling rate, bone matrix, and bone mineral deficiency; eventually, this can cause bone regeneration deficiency, osteoporosis, and osteomalacia (Liu et al., 2018). It is therefore essential that strategies are identified to effectively regulate the function of BMSCs for promoting osteogenic differentiation and bone regeneration.

In recent years, researchers have tried various approaches to boost stem cell function. Basic and clinical studies are increasingly investigating the promotion of osteogenic differentiation of BMSCs and the mechanisms involved, including traditional cytokines and related physical and chemical stimulation factors. Osteoblast growth peptide promotes osteogenic differentiation of BMSCs through the RhoA/ROCK pathway in a dose-dependent manner (Chen et al., 2011). Boron can promote the synthesis of osteogenic genes in the proliferation and differentiation of human BMSCs (Ying et al., 2011). The BMP-2 related peptides P24 (Lin et al., 2010) and simvastatin (Feng et al., 2020) also promote osteogenic differentiation and proliferation of BMSCs. BMPs are acidic proteins located in the bone matrix, and belong to the TGF-β superfamily. BMPs serve essential roles in skeletal development, bone formation, and MSC differentiation (Cai et al., 2021). Research has shown that fenofibrates induce PPARα and BMP2 expression to stimulate osteoblast differentiation; however, disruption in BMP signaling causes skeletal and vascular abnormalities (Miyazono, Kamiya and Morikawa 2010). In this context, a study showed BMP-2 and BMP-4 knockouts to be embryonically lethal in mice (Scarfì 2016). Thus, BMP2 serves an important role in inducing the osteogenic differentiation of MSCs (Toth et al., 2021).

In their study, Jian et al. (2013) applied 50, 100, and 200 μmol/L β-ecdysterone to human periodontal membrane stem cells (PDLSs) *in vitro* and confirmed that 200 μmol/L β-ecdysterone could effectively induce BMP-2 expression and osteogenic differentiation of periodontal membrane stem cells through the extracellular signal-regulated kinase pathway (Jian et al., 2013). However, it is unclear whether these positive effects of β-ecdysterone can also affect BMSCs and the specific molecular mechanisms involved and whether they can be applied to bone regeneration in animals or clinics.

β-ecdysterone is a polyhydroxylated steroid hormone, which is most abundant in insects and Anatidae plants. It is known as a phytoestrogen, because its chemical structure is similar to that of estrogen (Zou et al., 2015). β ecdysterone can not only stimulate protein synthesis (Tóth et al., 2008), promote carbohydrate and lipid metabolism (Catalán et al., 1985), control blood glucose level (Yoshida et al., 1971), inhibit cell apoptosis (Tang et al., 2018a), and improve intervertebral disc degeneration (Wen et al., 2019), but it also has good biocompatibility (Dai et al., 2017). Chinese herbal medicines such as *Achyranthe bidentata* have been used for centuries to treat osteoporosis and joint degeneration in China, and no side effects have been reported for hundreds of years. Studies have shown that β-ecdysterone can stimulate arthropod midgut stem cells (Smagghe et al., 2005) and induce osteogenic differentiation of mouse mesenchymal stem cells (Gao, Cai and Shi 2008). β-ecdysterone can regulate the proliferation and osteogenic differentiation of BMSCs by targeting estrogen receptors *in vivo* and plays an essential role in the process of bone regeneration (Abiramasundari et al., (2018). However, the specific signal transduction mechanism involved, the regulation mode of gene differential expression, and the optimal drug dose have not been discussed in depth. Therefore, a better understanding of the interactions and mechanisms between β-ecdysterone and BMSCs is expected to positively impact bone regeneration and formation.

This study aimed to explore whether β-ecdysterone can promote osteogenic differentiation and functionalization of BMSCs, enhancing their ability to promote *in situ* bone regeneration. Furthermore, it elucidated the potential signal transduction mechanism, differential regulation of gene expression, and appropriate dose of β-ecdysterone in promoting bone regeneration. During the in-vitro experiments, we treated MC3T3-E1 cells with β-ecdysterone to assess their biocompatibility and the osteogenesis-promoting effect. Cell Counting Kit-8 (CCK-8) was used to verify the excellent biocompatibility of β-ecdysterone. Immunohistochemical staining and quantitative polymerase chain reaction (q-PCR) were used to verify the excellent expression of alkaline phosphatase (ALP), collagen I, and other osteogenic proteins in MC3T3-E1 cells treated with β-ecdysterone. The alizarin red staining experiment further verified that the system could effectively form mineralized nodules from the extracellular matrix. Subsequently, MC3T3-E1 cells treated with different doses of β-ecdysterone were analyzed by gene sequencing and differential expression analysis of osteogenic-related genes. β-ecdysterone could effectively improve the replication and transcription of intracellular BMP-Smad signaling pathway genes in a dose-dependent manner. Finally, we added noggin, a BMP2 signaling pathway blocker, to explore any possible relationship between the BMP-2 signaling pathway, metabolism of BMSCs, and osteogenic differentiation after β-ecdysterone treatment; this was performed to evaluate the potential mechanism of enhanced bone regeneration. q-PCR and western blotting showed that β-ecdysterone significantly increased the expression of mRNA and proteins in the BMP2 signaling pathway, and this effect was inhibited by noggin, a BMP2 signaling pathway blocker. Furthermore, we established a rat model of femoral bone defect *in vivo* to evaluate the effect of β-ecdysterone on bone regeneration mediated by BMSCs. The animal experiments showed that at week 4 and 8 after surgery, rats injected intraperitoneally with 72 mg/kg of β-ecdysterone had a higher degree of gross bone tissue growth, bone mineral density, and degree of ossification in regenerated bone tissue at the site of the bone defect (as observed on immunohistochemical staining) than in the other groups. Overall, our data suggested that β-ecdysterone can mediate bone regeneration *via* the BMP2/Smad/Runx/Osterix signaling pathway. This study provides a new approach to the treatment of bone injury and degenerative diseases represented by bone defects and osteoporosis.

## Materials and Methods

### Materials

MC3T3-E1 cells (subclone 14) were purchased from Procell (Wuhan, China), induction medium (Cyagen, Guangzhou, China), α-modified Eagle medium (α-MEM, containing 4.5 g/L D-glucose, 25 mM HEPES), fetal bovine serum, 0.25% trypsin-EDTA, penicillin/streptomycin, and phosphate buffer saline (PBS) were purchased from Hyclone (Logan, UT, United States). Triton X-100, bovine serum albumin, and alizarin red S were purchased from Sangon Biotech (Shanghai, China). The RNeasy Mini Kit was purchased from Qiagen (Duesseldorf, Germany). The PrimeScript RT Master Mix and the TB Green Premix Ex Taq were purchased from Takara (Tokyo, Japan). The CCK-8, 4% paraformaldehyde, 10% cetylpyridinium chloride monohydrate, and β-ecdysterone were purchased from Solarbio (Beijing, China). The alkaline phosphatase assay kit and the goat anti-rabbit IgGDAB kit were purchased from Beyotime (Shanghai, China). Noggin, DAPI, SuperScript II reverse transcriptase, the RevertAid First Strand cDNA Synthesis Kit were purchased from Invitrogen (Thermo Fisher, United States), and 10% sodium dodecyl sulfate-polyacrylamide gel, cell lysis buffer, polyvinylidene fluoride membrane, and goat anti-rabbit antibody were purchased from Boster (Wuhan, China). All primary antibodies (type I collagen, osteopontin, BMP-2, Smad1/5, P-SMad1/5, Runx2, and Osterix) were purchased from Abcam. The animal anesthetic used was isoflurane (Jiangsu, Beikang, China), lidocaine (xylocaine 2%, Hebei Tiancheng, China).

### Cell Culture

MC3T3-E1 cells were cultured in α-MEM medium supplemented with 10% fetal bovine serum, 100 U/mL penicillin, and 100 g/ml streptomycin at 37°C in 5% CO_2_. The medium was replaced every 2–3 days. When cell fusion reached 80% (80% of the dish was covered by cells), 0.25% trypsin-EDTA was used for digestion, isolation, and passage culture. In our experiment, MC3T3-E1 of the third generation was used.

### Cell Proliferation

The CCK-8 assay was used to detect the biocompatibility of β-ecdysterone in MC3T3-E1 cells. MC3T3-E1 cells were incubated with 5 × 10^3^/well in 96-well plates, and 200 μl of α-MEM was added to each well. After incubation for 24 h, β-ecdysterone (Solarbio, Beijing, China) was added to 96-well plates at a final concentration of 0, 50, 100, 150, 200, and 250 μM. The cells were then incubated for 1–7 days. At each observation time point, cells were washed with PBS thrice; 10 μl CCK-8 solution and 100 μl fresh α-MEM medium were added to each well and then incubated at 37°C for 1 h. The absorbance was measured at 460 nm using a microplate reader (Thermo Fisher United States).

### Alkaline Phosphatase Activity

ALP activity was measured using an ALP Assay Kit (Beyotime, Shanghai, China). MC3T3-E1 cells were cultured in 6-well plates at 2 × 10^4^ cells/well. When the degree of cell fusion exceeded 60% (60% of the dish was covered by cells), β-ecdysterone was added to the medium at final concentrations of 0, 100, 150, and 200 μM with induction medium. After 3 or 7 days of culture, the cells were washed with PBS and fixed with 4% paraformaldehyde for 15 min. Triton X-100 was used to rupture the cell membranes and cell proteins were extracted by centrifugation at 12,000/min. According to the manufacturer’s instructions for the ALP kit, extracts from the control, standard, and experimental groups were transferred to 96-well plates, at volumes of 4, 8, 16, 24, 32, and 40 μl, respectively. The protein concentration was normalized before transfer. Detection buffer and chromogenic substrate were added to achieve a total volume 100 μl, and the reaction system was incubated in darkness at 37°C for 10 min. A stop buffer was added to each well to stop the reaction and the absorbance at 405 nm was measured using a microplate reader.

### Alizarin Red S Staining

To test the mineralization ability of MC3T3-E1 cells induced by β-ecdysterone, calcium nodules were detected by alizarin red S staining. MC3T3-E1 cells were incubated at a density of 1 × 10^4^ cells/cm^2^ in a dish (*φ* = 30 mm) and incubated in a medium containing 150 μM of β-ecdysterone or induction medium. The medium and β-ecdysterone were replaced every 3 days. On day 21, the cells were washed with PBS and fixed with 4% paraformaldehyde for 30 min; they were then stained with a 1% alizarin red S solution for 20 min. Decolorization was performed with 10% cetylpyridinium chloride monohydrate for 20 min, and absorbance was measured at 595 nm for quantitative analysis.

### Immunocytochemical Staining and Immunofluorescence Staining

To investigate the effect of osteogenic-related protein expression in MC3T3-E1 cells treated with β-ecdysterone, MC3T3-E1 cells were incubated with 1.5 × 10^4^ cells/well in 6-well plates and cultured in standard medium, medium containing 150 μM/L β-ecdysterone, and induction medium. After 14 days of culture, the cells were washed thrice with PBS and fixed in 4% paraformaldehyde solution at room temperature for 15 min. The cells were washed again with PBS and treated with 0.1% Triton X-100 for 15 min. The cells were then incubated in a 5% bovine serum albumin solution at 37°C for 1 h. After washing the cells thrice with PBS, either osteopontin antibody (1:200) or secondary antibody and hematoxylin were added; the cells were then incubated at 4°C overnight, followed by incubation with goat anti-rabbit IgG at room temperature for 30 min. The DAB horseradish peroxidase chromogenic kit was used to detect osteopontin expression in cells. The nuclei were then stained with hematoxylin for 3 min and osteopontin staining was observed under an inverted microscope (Leica Microsystems CMS, Wetzlar, Germany).

Immunofluorescence staining was used to detect type I collagen expression in cells treated with different concentrations of β-ecdysterone. MC3T3-E1 cells were incubated with 1 × 10^4^ cells/well in 6-well plates and cultured in a medium containing 0, 100, 150, and 200 μM/L of β-ecdysterone; alternatively, they were cultured in induction medium. In addition, noggin (0.5 mg/ml) was added in the control group. After 10 days of induction culture, the cells were fixed at room temperature with 4% paraformaldehyde for 30 min, washed thrice with PBS, and treated with Triton X-100 for 15 min to rupture the cell membranes. The cells were washed again with PBS and blocked at room temperature with 10% normal goat serum for 1 h. The primary antibody of type I collagen was added followed by incubation overnight at 4°C; this was followed by an appropriate dose of fluorescent secondary antibody at room temperature for 30 min. The cells were rewashed thrice with PBS and stained with DAPI nuclear stain (0.1 mg/ml; Sigma-Aldrich, St. Louis, MO, United States) for 15 min. A confocal laser scanning microscope (Olympus, Tokyo, Japan) was used to observe the distribution of type I collagen and the fluorescence intensity of type I collagen in the cytoplasm was quantified by ImageJ software (Wayne Rasband, NIH, United States). The determination was repeated thrice in each group.

### RNA Sequence Analysis and Gene Enrichment Analysis

To observe and compare gene expression in MC3T3-E1 cells treated with β-ecdysterone, we performed RNA sequencing of the samples. Third generation MC3T3-E1 cells were incubated in a petri dish measuring 10 cm in diameter in a standard medium containing 0, 100, 150, and 200 μM of β-ecdysterone for 5 and 10 days. When the number of cells reached 3 × 10^6^–5 × 10^6^ cells/well, the RNA was extracted by TRIzol (Qiagen) lysis (*n* = 3). After the qualified samples were detected, the TruSeq RNA sample preparation kit was used to construct a sequencing gene bank (Illumina). First, magnetic beads with oligo (dT) enriched eukaryotic mRNA were used, and the mRNA was randomly interrupted by fragmentation buffer. Second, using mRNA as a template, cDNA was synthesized by reverse transcription of RNA using SuperScript II reverse transcriptase (Invitrogen) and cDNA was purified using AMPure XP beads. Third, the purified double-stranded cDNA was repaired, a-tailed, and sequenced. Finally, AMPure XP Beads were used for fragment size selection, and cDNA libraries were obtained by PCR enrichment. After the library was constructed, sequencing was performed using the Illumina platform and bioinformatics analysis was performed at Qingdao Bioscience and Technology Co., Ltd.

### Real-Time-qPCR

Real-time PCR (RT-qPCR) was performed to further verify the results of gene sequencing and the impact of the β-ecdysterone on bone regeneration. MC3T3-E1 cells were incubated in 6-well plates at a density of 5 × 10^5^ cells/well. When the cell density exceeded 60% (60% of the dish was covered by cells), induction medium with 150 μM of β-ecdysterone was added in the experimental group, and noggin (0.5 mg/ml) was added in the control group. On days 7 and 10, total RNA was extracted from MC3T3-E1 cells using TRIzol reagent and cDNA was synthesized using the RevertAid First Strand cDNA Synthesis Kit. The cDNA concentration was normalized before transfer. The RT q-PCR was performed using FastStart Universal SYBR Green Master (Rox) (Roche, Germany). The BMP2, Runx2, Osterix, Col I, and GAPDH primer sequences are shown in Table 1. Relative gene expression was calculated using the 2^−ΔΔCT^ method and all experiments were repeated thrice.

**TABLE 1:** RT-qPCR primer sequences.

Gene	Primer sequences
Bmp-2	Forward: 5′-CAC​GAG​AAT​GGA​CAT​GCC​C-3′
	Reverse: 5′-GCT​TCA​GGC​CAA​ACA​TGC​TG-3′
Runx2	Forward: 5′-GCT​GTT​GTG​ATG​CGT​ATT​CCC-3′
	Reverse: 5′-TGA​ACC​TGG​CCA​CTT​GGT​TT-3′
Osterix	Forward: 5′-GAT​GGC​GTC​CTC​TCT​GCT​TG-3′
	Reverse: 5′-AAT​GGG​CTT​CTT​CCT​CAG​CC-3′
Collagen I	Forward: 5′-AAG​GCT​CCC​CTG​GAA​GAG​AT-3′
	Reverse: 5′-CAG​GAT​CGG​AAC​CTT​CGC​TT-3′
GAPDH	Forward: 5′-TCC​ATG​ACA​ACT​TTG​GTA​TCG-3′
	Reverse: 5′-TGT​AGC​CAA​ATT​CGT​TGT​CA-3′

### Protein Electrophoresis Analysis

MC3T3-E1 cells were incubated in 6-well plates at a density of 5 × 10^5^ cells/well. Cells cultured in a medium containing different concentrations (0–200 μM) of β-ecdysterone and noggin (0.5 mg/ml) were included in the experimental and control groups, respectively. After 7 days of induction culture, proteins were extracted with cell lysis buffer; the protein concentration was normalized before transfer. Proteins denatured in equal amounts from different samples were separated by electrophoresis on 10% sodium dodecyl sulfate-polyacrylamide gel (Beyotime) and then transferred to a polyvinylidene fluoride membrane. After the protein transfer membrane was enclosed in blocking buffer (Tris-buffered saline containing 0.1% Tween 20 and 5% fat-free milk) for 1 h, it was incubated with primary antibody at 4°C overnight. The goat anti-rabbit antibody (Boster) was then incubated at 37°C for 2 h. The ChemiDoc XRS + chemiluminescence detection system (BIO-RAD) was used for observation and the strip strength was analyzed using ImageJ software. The primary antibodies used were BMP-2 (1:1,000, Abcam), Smad1/5 (1:1,000, Abcam), P-SMad1/5 (1:1,000, Abcam), Runx2 (1:1,000, Abcam), and Osterix (1:1,000, Abcam). All experiments were repeated thrice.

### Rat Model of Bone Defects

The animal experiments were approved by the Research Ethics Committee of the Affiliated Hospital of the North Sichuan Medical College (2021–26). Fifteen male Sprague Dawley rats (6–8 weeks old, weighing approximately 200 g) were selected for the animal experiments. The rat model of bone defects (Yan et al., 2019; Li and Helms 2021) was constructed after anesthetizing with inhalational isoflurane (for animals); an anesthesia ventilator was used for maintenance. The anesthesia protocol was as follows: the rat was placed in a closed glass container and anesthetized with 2.5% isoflurane in 30% oxygen (Schubert et al., 2012). During anesthesia induction, the inhalational concentration of isoflurane was raised to 1.5%–3.0% within 7–10 min. Once the four limbs of the rat were limp and no pain reflex was elicited, continuous anesthesia was initiated, oxygen inhalation was maintained *via* a mask, and the concentration of isoflurane was maintained at 1%–2.5%. Incisions were performed after subcutaneous infiltration of lidocaine for local anesthesia. After the operation, the rats were allowed to breathe air freely until they were fully awake.

The specific method of surgery was as follows: after skin preparation, the medial condyle of the femur was exposed and a bone defect measuring 3 mm in diameter and 4 mm in depth was created using a K wire of 3.0 mm in diameter with a slow-speed electric drill; the site was irrigated using ice-cold saline solution to avoid thermal necrosis. The operative region was then sutured layer by layer. The sham operation group (*n* = 5) only received anesthesia and skin surgery, with no damage to the femur condyle. Rats with bone defects (*n* = 10) were categorized into 2 groups to receive intraperitoneal injections of 0 mg/kg of PBS (*n* = 5) and 72 mg/kg of β-ecdysterone (*n* = 5), respectively, every 3 days. At 4 and 8 weeks after surgery, the mice were over-anesthetized to death. The femur was harvested and fixed with a 4% paraformaldehyde solution for inspection.

### *In Vivo* Toxicology Studies

The animals were segregated into three groups. The group of animals administered 72 mg/kg of intraperitoneal β-ecdysterone for 4 weeks comprised the experimental group; the group administered PBS served as the control group. The untreated mice served as the sham group. After 4 weeks of treatment, the animals were sacrificed by over-anesthesia. The liver and kidney tissues of rats were sectioned and stained with hematoxylin and eosin to observe toxicities *in vivo*.

### Micro-Computed Tomography Analysis

All samples collected from rat models with femoral condylar defects were fixed in 4% paraformaldehyde at room temperature for 24 h. Micro-computed tomography (CT) (u-ct80, SCANCO, Switzerland) was used to test the samples (Clark and Badea 2021). Three-dimensional reconstruction was performed using the processed images (Scanco^®^ software) and the bone volume, trabecular thickness, and bone mineral density of each group were detected and analyzed.

### Immunohistochemical Analysis

All femoral condyle samples were decalcified and embedded in paraffin after micro-CT analysis. A 5-μm-thick tissue section was analyzed at the bone defect site for histomorphological analysis and the detection of new site-specific proteins of bone tissue (including BMP2 and Runx2). The sections were then stained with hematoxylin and eosin for histochemistry. Images of the histological specimen were obtained using a microscope (Eclipse E800; Nikon, Japan).

### Statistical Analysis

Statistical analysis was performed using SPSS 23.0 (IBM Corp., Armonk, NY, United States) and Graphpad Prism 9 (GraphPad Software, United States). The independent sample *t*-test was used to evaluate statistical differences between the two groups and one-way analysis of variance (ANOVA) was used for multiple data groups. Data have been presented as means ± standard deviation. *p* < 0.05 was considered statistically significant.

## Results

### β-Ecdysterone Promoted the Proliferation of MC3T3-E1 Cells *In Vitro*

**FIGURE 1:**
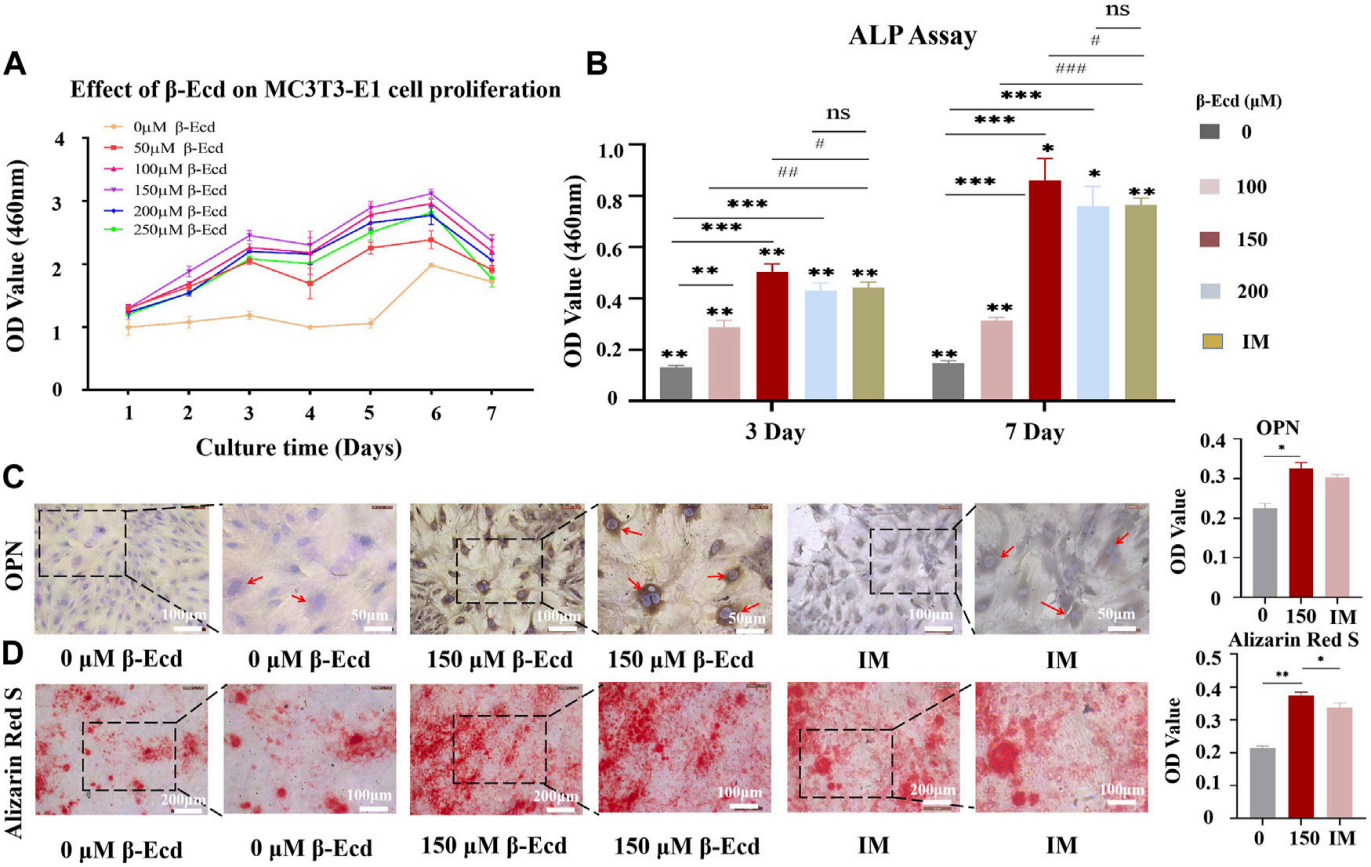
Osteogenic effect of β-Ecd on MC3TE-E1 cells *in vitro*. **(A)** The effects of β-Ecd on the proliferation of MC3TE-E1cells. MC3TE-E1 cells were cultured with medium containing 0 μM β-Ecd (control group) and 50–250 μM β-Ecd (experimental group) and evaluated by the CCK-8 assay. The cell proliferation rate at 1–7 days is shown (*n* = 5). β-Ecd could increase the cell proliferation rate to different degrees, among which 150 μM β-Ecd was the most significant. **(B)** Alkaline phosphatase activity assay. After culturing MC3TE-E1 for 3 and 7 days with medium containing 0 μM β-Ecd (control group) and 100–200 μM β-Ecd (experiment group) or induction medium, intracellular ALP activity was measured. The results showed that the activity of ALP in cells treated with β-Ecd was higher than that of the control group and the most significant increase occurred at 150 μM β-Ecd, there was no significant difference 200 μM β-Ecd group compared with the IM group. The “*” stands for the control group (0 μM) was compared with each experimental group, *p < 0.05, **p < 0.01, ***p < 0.001. The “^#^” stands for the IM group was compared with each experimental group, ^#^ *p* < 0.05, ^##^ *p* < 0.01, ^###^ *p* < 0.001. **(C)** Immunocytochemical staining to detect the expression of osteopontin in MC3TE-E1, after treating MC3TE-E1 with 0 μM β-Ecd (control group) and 150 μM β-Ecd (experimental group) or induction mediun for 14 days, the immunochemistry of osteopontin in cells. The staining results showed that the expression level of the experimental group was significantly higher than that of the control group, there was no significant difference compared with the IM group. The yellow-brown particles in the cytoplasm are osteopontin. **(D)** Alizarin Red S staining to detect the formation of calcified nodules by MC3TE-E1 and its extracellular matrix. After 21 days of treatment with 0 μM β-Ecd (control group) and 150 μM β-Ecd (experimental group) or induction medium, the results of Alizarin Red S staining showed that the formation of calcium nodules in the experimental group was significantly higher than in the control group, there was no significant difference compared with the IM group. In red is the calcium nodule. ImageJ software was used to measure the relative expression values in the figure, and three independent experiments were carried out, and the data were expressed as *X ± SD*; *p < 0.05, **p < 0.01, ***p < 0.001.

To understand the biocompatibility of β-ecdysterone on MC3T3-E1 cells, we treated MC3T3-E1 cells with different concentrations of β-ecdysterone (0, 50, 100, 150, 200, and 250 μM); the CCK-8 assay was used to detect its effect on cell proliferation. The results showed that β-ecdysterone did not significantly inhibit the proliferation of MC3T3-E1 cells at different concentrations, but showed different proliferative abilities (Table 2; Figure 1A). Cell proliferation activity gradually increased with an increase in drug concentrations from 0 to 150 μM; however, this activity did not continue to increase when drug concentrations increased from 150 to 250 μM. Therefore, we treated cells with β-ecdysterone concentrations of 100, 150, and 200 μM in subsequent experiments.

**TABLE 2:** Effects of β-ecdysterone on the proliferation of MC3T3-E1 cells (*OD value*, *X ± SD*).

Culture (day)	β-ecdysterone concentration (μM/L)					
	0	50	100	150	200	250
1	1.00 ± 0.13	1.30 ± 0.03**	1.29 ± 0.07**	1.30 ± 0.06**	1.23 ± 0.11*	1.20 ± 0.01*
2	1.08 ± 0.09	1.64 ± 0.05***	1.69 ± 0.03***	1.88 ± 0.08***	1.54 ± 0.04***	1.54 ± 0.02***
3	1.19 ± 0.06	2.04 ± 0.05***	2.26 ± 0.06***	2.45 ± 0.08***	2.20 ± 0.01***	2.08 ± 0.05***
4	1.00 ± 0.03	1.69 ± 0.24*	2.18 ± 0.34***	2.30 ± 0.12***	2.16 ± 0.17***	2.01 ± 0.27***
5	1.06 ± 0.08	2.75 ± 0.09***	2.78 ± 0.21***	2.89 ± 0.07***	2.65 ± 0.11***	2.50 ± 0.12***
6	1.98 ± 0.03	2.89 ± 0.14***	2.96 ± 0.07***	2.94 ± 0.07***	2.77 ± 0.15***	2.82 ± 0.06***
7	1.72 ± 0.03	1.91 ± 0.05**	2.20 ± 0.26**	2.37 ± 0.09*	2.06 ± 0.09*	1.77 ± 0.14**

Values are expressed as means *X ± SD* (*n* = 5). The control group (0 μM) was compared with each experimental group. *p < 0.05, **p < 0.01, ***p < 0.001, NS, no significance.

### β-Ecdysterone Enhance Osteogenic Differentiation of MC3T3-E1 Cells *In Vitro*

ALP is an osteoblast marker secreted at the beginning of osteogenic differentiation. To explore the role of β-ecdysterone in promoting osteogenic differentiation of MC3T3-E1 cells, we examined ALP activity in MC3T3-E1 cells. The results showed that intracellular ALP activity increased after treatment with different concentrations of β-ecdysterone (0, 100, 150, and 200 μM) for 3 and 7 days and the effect was dose- and time-dependent. In addition, 150 μM β-ecdysterone induced the most significant increase in ALP activity, which was significantly higher on day 7 than on day 3 (Table 3; Figure 1B).

**TABLE 3:** ALP activity detection (*n* = 6).

Time (days)	β-ecdysterone (μM)	‾*X ± SD* (OD value)	DEA/mg
3	0	0.132 ± 0.01	48.73
	100	0.287 ± 0.03**^/##^	129.51
	150	0.503 ± 0.03***^/#^	241.37
	200	0.429 ± 0.03***^/NS^	202.79
	IM	0.442 ± 0.02	211.26
7	0	0.146 ± 0.01	56.5
	100	0.314 ± 0.01***^/###^	143.49
	150	0.860 ± 0.08***^/#^	426.23
	200	0.759 ± 0.08***^/NS^	373.93
	IM	0.766 ± 0.03	378.12

Values are expressed as means *X ± SD* (*n* = 6). The “*” stands for the control group (0 μM), which was compared with each experimental group. *p* < 0.05, **p < 0.01, ***p < 0.001. The “#” stands for the IM group, which was compared with each experimental group. ^#^ *p* < 0.05, ^##^ *p* < 0.01, ^###^ *p* < 0.001. ALP, alkaline phosphatase activity; DEA, diethanolamine enzyme activity unit.

Osteopontin (OPN) is an osteogenic marker secreted by osteoblasts in the middle and late stages of osteogenic differentiation. Immunocytochemical staining was performed on MC3T3-E1 cells treated with β-ecdysterone (0 and 150 μM) to investigate whether it also promoted MC3T3-E1 cells in the middle and late stages of osteogenic differentiation. The results showed that the brownish-yellow granules in the cytoplasm of the experimental group were significantly higher than those of the control group. There was no significant difference between the 150 μM β-ecdysterone and IM groups (Figure 1C). However, the nucleus was observed in the cells of the antibody controls (OPN group) in our study; no OPN expression was observed in the cytoplasm (Supplementary Figure S1).

During osteogenesis, osteoblasts undergo proliferation and gradually differentiate into osteocytes. Calcium salts are deposited in bone cells before they form bone tissue. The cells then fuse, mineralize, and form mineralized nodules. In our study, alizarin red staining was used to compare cells cultured for 21 days to investigate the effect of β-ecdysterone on mineralized nodule formation in MC3T3-E1 cells at the end stage of differentiation. The results showed that MC3T3-E1 cells cultured in osteoblast induction medium under β-ecdysterone intervention had more mineralized nodules than those cultured in osteoblast induction medium alone (Figure 1D). These results suggest that β-ecdysterone can enhance osteogenic differentiation of MC3T3-E1 cells and improve their ability to form bone tissue *in vitro*.

### Gene Sequencing Analysis of MC3T3-E1 Cells Treated With β-Ecdysterone

**FIGURE 2:**
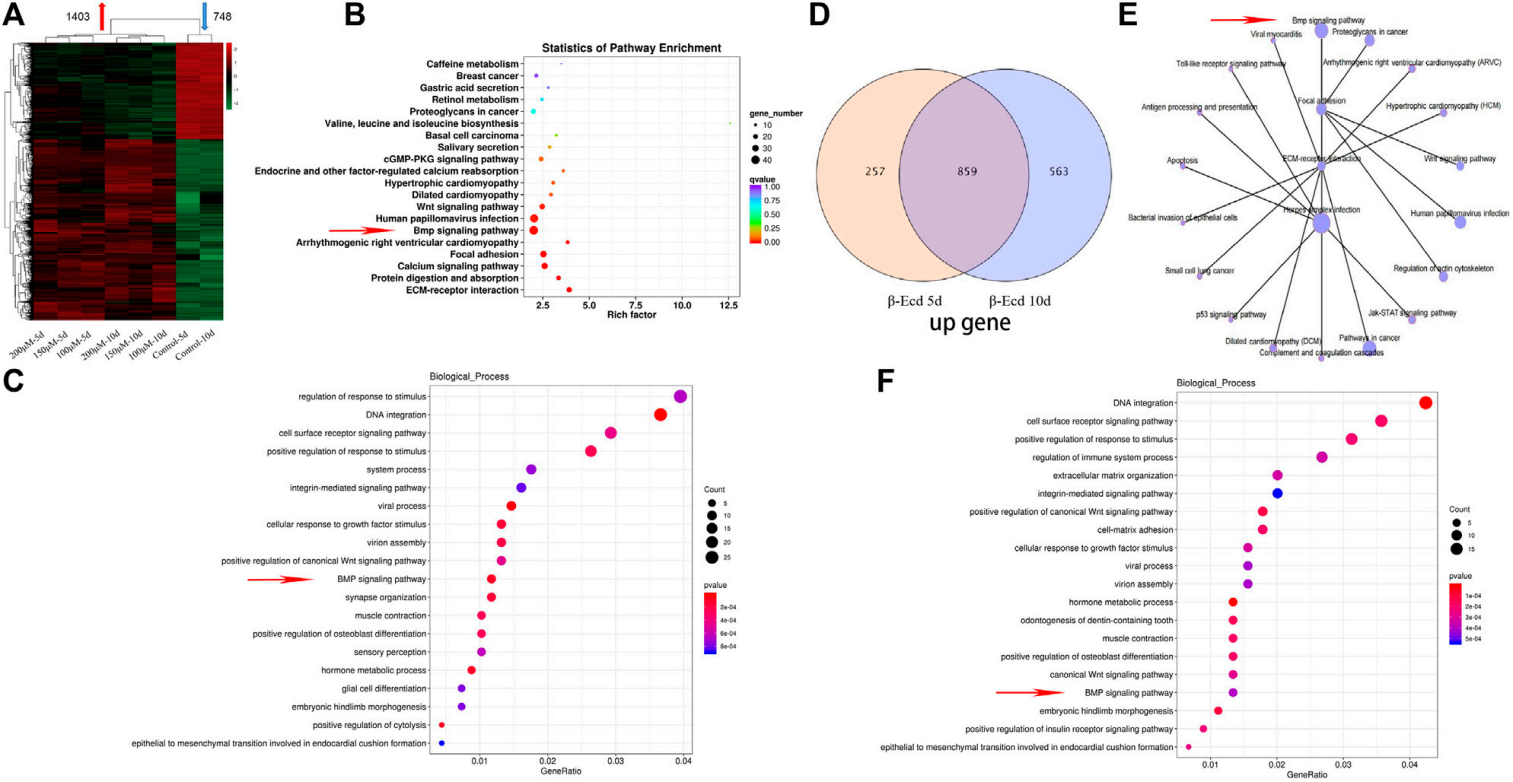
Intracellular RNA was extracted at 5 and 10 days of culture, and transcriptome RNA sequencing analysis was performed. **(A)** Cluster analysis of differentially expressed genes |log2 ratio| > 1 and FDR < 0.05 in the 0–200 μM β-Ecd group compared to 5 and 10 days. **(B)** Differential gene enrichment of the KEGG signaling pathway. **(C)** Analysis of cell biological processes of differential gene enrichment biological processes. **(D)** Venn analysis of differentially expressed genes. **(E)** KEGG signaling pathway of mRNAs associated with osteogenic differentiation. **(F)** Analysis of the cell biology enrichment process of 859 genes upregulated in the Venn analysis.

To understand the specific effects of β-ecdysterone on nucleic acid transcription and expression in MC3T3-E1 cells, we used mRNA-seq to study the gene expression of MC3T3-E1 cells treated with β-ecdysterone at days 5 and 10. As shown in Figure 2A, among all detected mRNAs, 29,583 genes were found to be involved in gene expression analysis compared to the known mouse genome. In the experimental group, 1403 and 748 genes were up- and down-regulated, respectively; log2 > 1 and Q < 0.05 were established as indicators of significant difference. Further analysis of biological processes enriched by these differentially expressed genes using Kyoto Encyclopedia of Genes and Genomes clustering analysis and Gene Ontology functional enrichment analysis showed that genes of the BMP signaling pathway were among the top 20 upregulated genes; the differences were significant (Figures 2B,C). We analyzed the intersection of gene expression in MC3T3-E1 cells treated with β-ecdysterone for 5 and 10 days using Venn diagrams. A total of 1310 genes were up- or downregulated, including 859 upregulated genes (Figure 2D). We analyzed these 859 upregulated genes (Figure 2F) and found that osteogenesis-related genes (BMP and Wnt) were almost all upregulated; related signal transduction genes, including those associated with DNA integration and cell membrane receptors, were significantly upregulated. Kyoto Encyclopedia of Genes and Genomes analysis of signaling pathways of osteogenic target genes revealed that genes of the BMP, Wnt, and extracellular matrix-receptor interaction signaling pathways were enriched (Figure 2E).

### β-Ecdysterone Induced the Expression of BMP-2, Runx2, and Osterix mRNA in MC3T3-E1 Cells *In Vitro*

**FIGURE 3:**
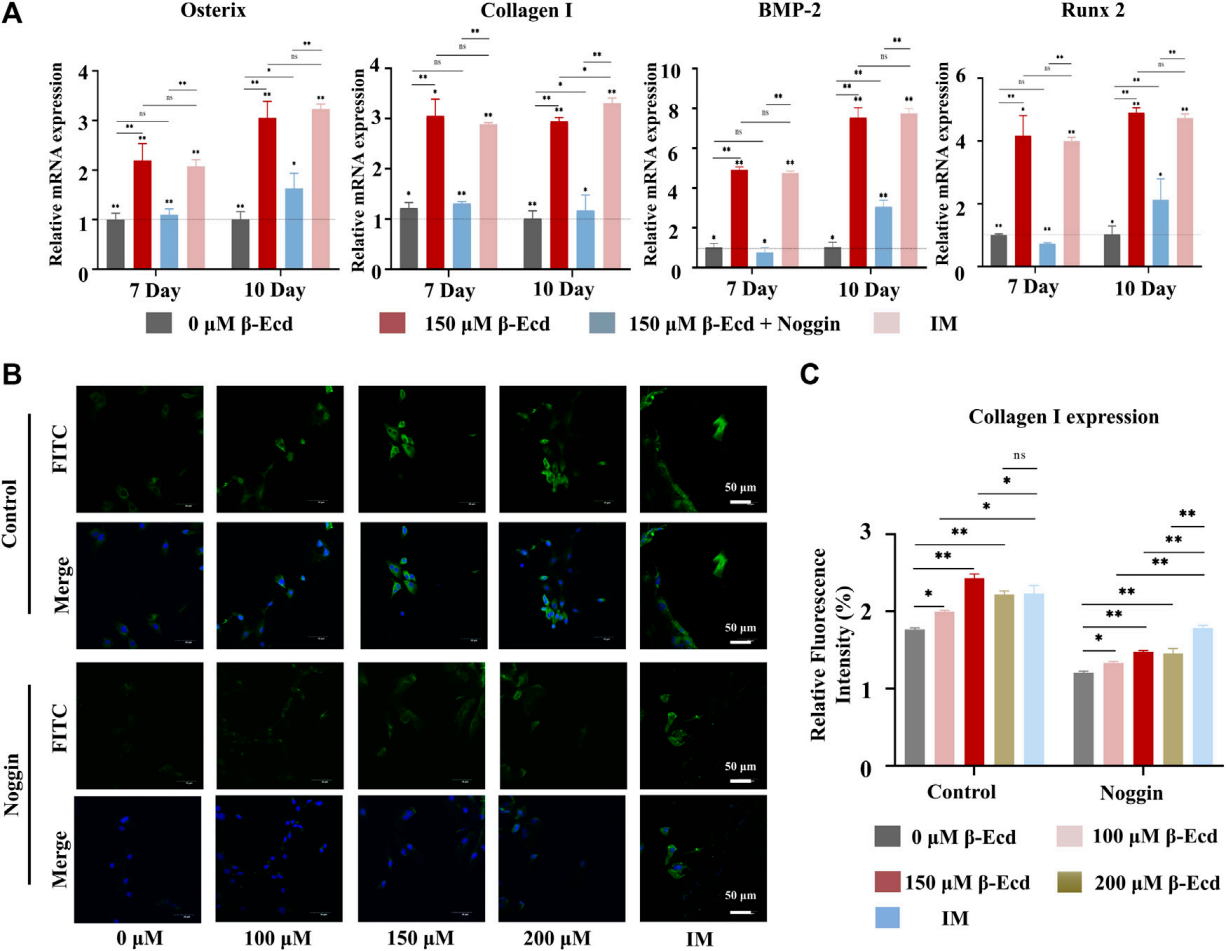
β-Ecd promotes the expression of osteogenic proteins and genes. **(A)** The relative expression levels of osteogenic differentiation-related genes in MC3TE-E1 treated with induction medium, β-Ecd and noggin were determined by RT-qPCR. The results showed that β-Ecd could significantly increase the expression of osterix mRNA, Collagen I mRNA, BMP-2 mRNA, and Runx2 mRNA in cells; however, this effect could be inhibited by exposure to noggin. There was no significant difference compared with the IM group and 150 μM β-Ecd group. The experiments were repeated three times and the results were normalized by the expression level of GAPDH. *p < 0.05, **p < 0.01. **(B,C)** Immunofluorescence (IF) staining to detect type I collagen expression in MC3T3-E1. After treating MC3TE-E1 with 0 μM β-Ecd (control group) and 100–200 μM β-Ecd (experimental group) or iduction medium for 10 days, the IF results showed that the expression of type I collagen in the experimental group increased significantly compared to the control group, but this effect could be explained by inhibition of noggin, type I collagen is shown in green and nuclei are shown in blue. There was no significant difference compared with the IM group and 150 μM β-Ecd group. ImageJ software was used to measure the relative expression values in the figure, and three independent experiments were carried out, *p < 0.05, **p < 0.05.

BMP-2 has been shown to induce osteoblast differentiation rapidly and effectively *in vitro*. Furthermore, BMP-2 plays a vital role in bone formation and remodeling. Using mRNA sequencing analysis, our study found that β-ecdysterone enhanced the enrichment of genes from the osteogenic signaling pathway, including the BMP signaling pathway. To investigate the effect of genes of the BMP-2 signaling pathway on β-ecdysterone-mediated (0/150 μM) osteogenic differentiation of MC3T3-E1 cells, we performed RT-qPCR to measure the expression of osteogenic-related genes. The RT-qPCR results showed that β-ecdysterone significantly increased the expression of BMP-2, Runx2, Col I, and Osterix (Figure 3A). To further verify the involvement of BMP-2 in β-ecdysterone induced osteogenic differentiation, we used the BMP-2 receptor antagonist noggin to block BMP-2 signaling in MC3T3-E1 cells. Noggin treatment of MC3T3-E1 cells reduced the expression of BMP-2, Runx2, Osterix, and Collagen I mRNA. These data suggested that the BMP-2 signaling pathway plays a significant role in osteogenic differentiation of MC3T3-E1 cells mediated by β-ecdysterone (Figure 3A).

### β-Ecdysterone Regulated Osteogenic Differentiation of MC3T3-E1 Cells Through the BMP-2/Smad/Runx2/Osterix Signaling Pathway

**FIGURE 4:**
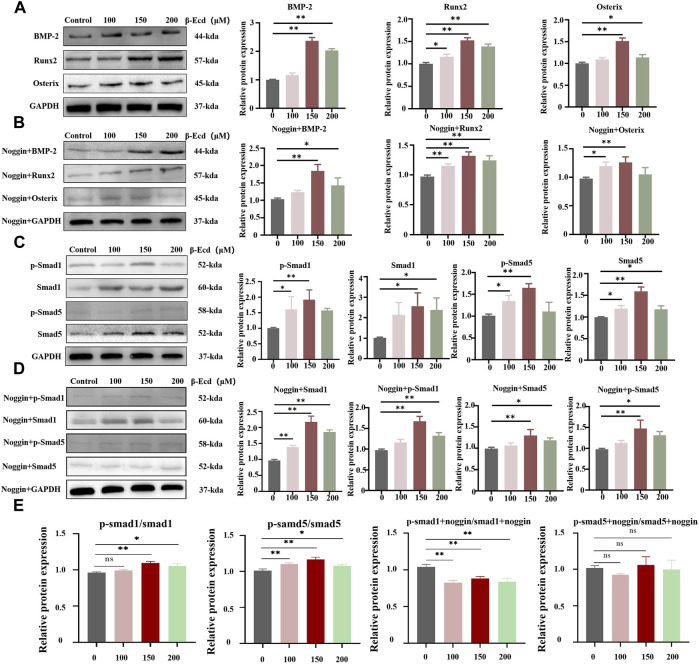
Western blotting detection of BMP-2, Smad1/5, p-Smad1/5, Runx2 and Osterix protein expression levels in MC3TE-E1 cultured with β-Ecd and noggin. **(A,B)**, BMP-2, Runx2, Osterix protein expression levels, **(C,D)**, Smad1/5, phosphorylated (p)-Smad1/5 protein expression levels. **(E)** the ratio of the protein to phos-protein, β-Ecd could increased the ratio of the protein to phos-protein, but, the noggin inhibit this. ImageJ software was used to calculate the densitometric analysis of the protein bands (*N* = 3; *p < 0.05, **p < 0.001).

Western blotting was used to detect the expression of the BMP-2, Smad1/5, phosphorylated (p)-Smad1/5, RUNX2, and osterix proteins induced by β-ecdysterone. We confirmed the role of the BMP-2/SMAD/RUNX2/Osterix pathway in β-ecdysterone-mediated osteogenic differentiation of MC3T3-E1 cells. Our data showed that β-ecdysterone significantly increased intracellular BMP-2, Smad1/5, p-Smad1/5, Runx2, and osterix proteins; the ratio of protein to phos-protein also increased significantly, with the most significant increase observed at 150 μM (Figure 4). Furthermore, there was no effect on the levels of the GAPDH protein. The protein expression of BMP-2, Smad1/5, phosphorylated (p)-Smad1/5, Runx2, and Osterix were significantly decreased in MC3T3-E1 cells treated with noggin. Therefore, these data suggested that the BMP-2/Smad/Runx2/Osterix signaling pathway is involved in the regulation of osteogenic differentiation of MC3T3-E1 cells by β-ecdysterone.

Immunofluorescence was used to detect the expression of collagen I in the cytoplasmic region and the effect induced by exposure to noggin. The results showed that collagen I immunofluorescence aggregation differed significantly between the experimental and control groups (*p* < 0.05). Collagen I expression was most significant in the experimental group when the concentration of β-ecdysterone was 150 μM. In contrast, collagen I expression in cells treated with noggin was generally decreased (*p* < 0.05), as shown in Figures 3B,C. These data suggest that noggin may inhibit the positive regulation of collagen I expression in MC3T3-E1 cells.

### Therapeutic Effect of β-Ecdysterone on Femoral Bone Defects in Rats

The bone defect model was established in this study by drilling the femoral condyle in rats. According to the results of the *in vitro* experiment, the rats were divided into three groups: the control group (0 mg/kg of β-ecdysterone was injected intraperitoneally), experimental group (72 mg/kg of β-ecdysterone was injected intraperitoneally), and the sham operation group. Rats were injected intraperitoneally with the corresponding drugs every 3 days after surgery and were sacrificed at the eighth week for micro-CT scanning, reconstruction, and immunohistochemical staining.

**FIGURE 5:**
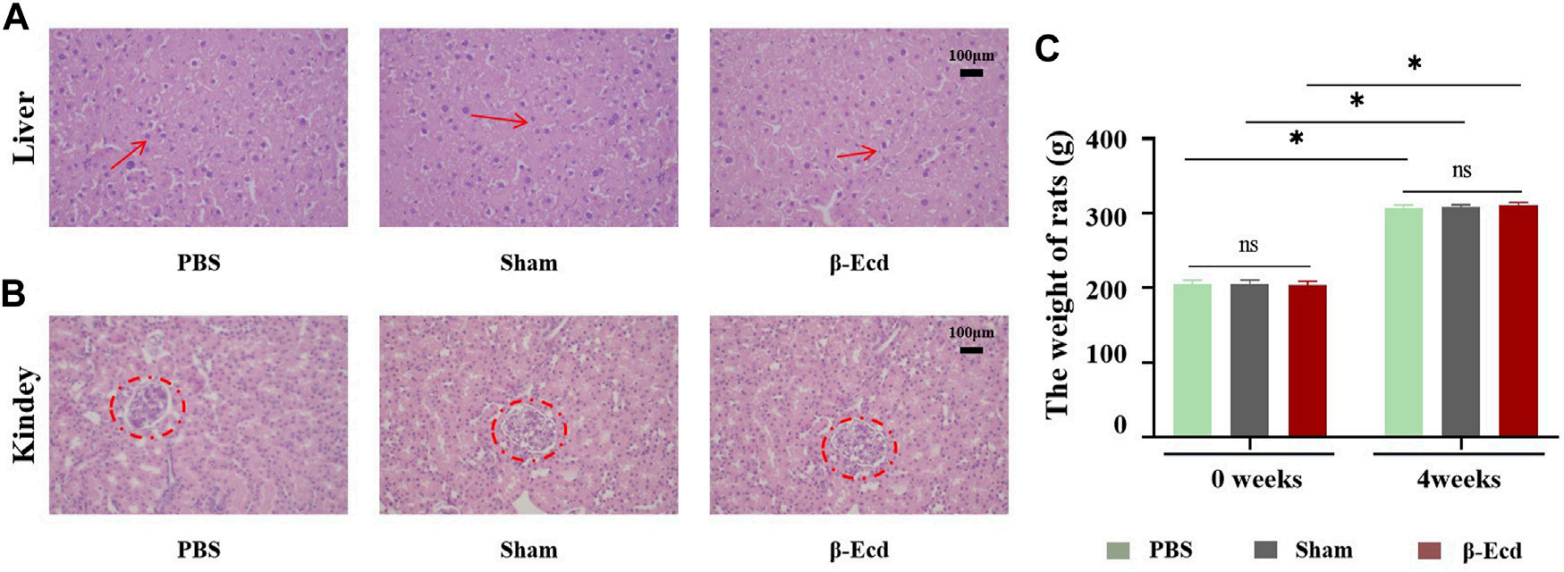
Effect of β-ecdysterone on tissue architecture of rats 4 weeks after surgery was compared with that before surgery. **(A)** Depicts the hematoxylin and eosin stained section of liver from control rat, sham rat and rat treated with 92 mg/kg β-ecdysterone showing no signs of hepatotoxicity (shown in arrow). **(B)** Depicts the hematoxylin and eosin stained section of kidney from control rat, sham rat and rat treated with 92 mg/kg β-ecdysterone showing no signs of nephrotoxicity (shown in circle). **(C)** 4 weeks after surgery, the weight of rats in different groups increased respectly, and β-ecdysterone showing no signs of weight loss. *p < 0.05 and NS, no significant.

Table 4 and Figure 5 show the results of weight and histopathology analysis of different tissues in the control, sham, and experimental groups. Tissue sections of the liver and kidney of rats treated with 72 mg/kg of β-ecdysterone for 4 weeks showed no signs of abnormality and toxicity, respectively. This further confirmed that β-ecdysterone did not exert any undesirable toxic effects on the animals at low doses.

**TABLE 4:** The weight of rats in different groups (0 and 4 weeks after surgery; *n* = 5).

Time (days)	PBS group	Sham group	β-ecdysterone group	Statistics (F, *p*)
0 weeks	204.7 ± 2.48	204.4 ± 2.96	203.9 ± 2.76	F = 0.13, *p* = 0.89
4 weeks	306.9 ± 4.86	308.5 ± 3.59	311.0 ± 4.87	F = 1.66, *p* = 0.23
Statistics (*t*, *p*)	*t* = 33.6, *p* < 0.01	*t* = 35.9, *p* < 0.01	*t* = 39.8, *p* < 0.01	

Values are expressed by means *X* ± *SD* (*n* = 5). **p < 0.01, ***p < 0.001, NS, no significance.

**FIGURE 6:**
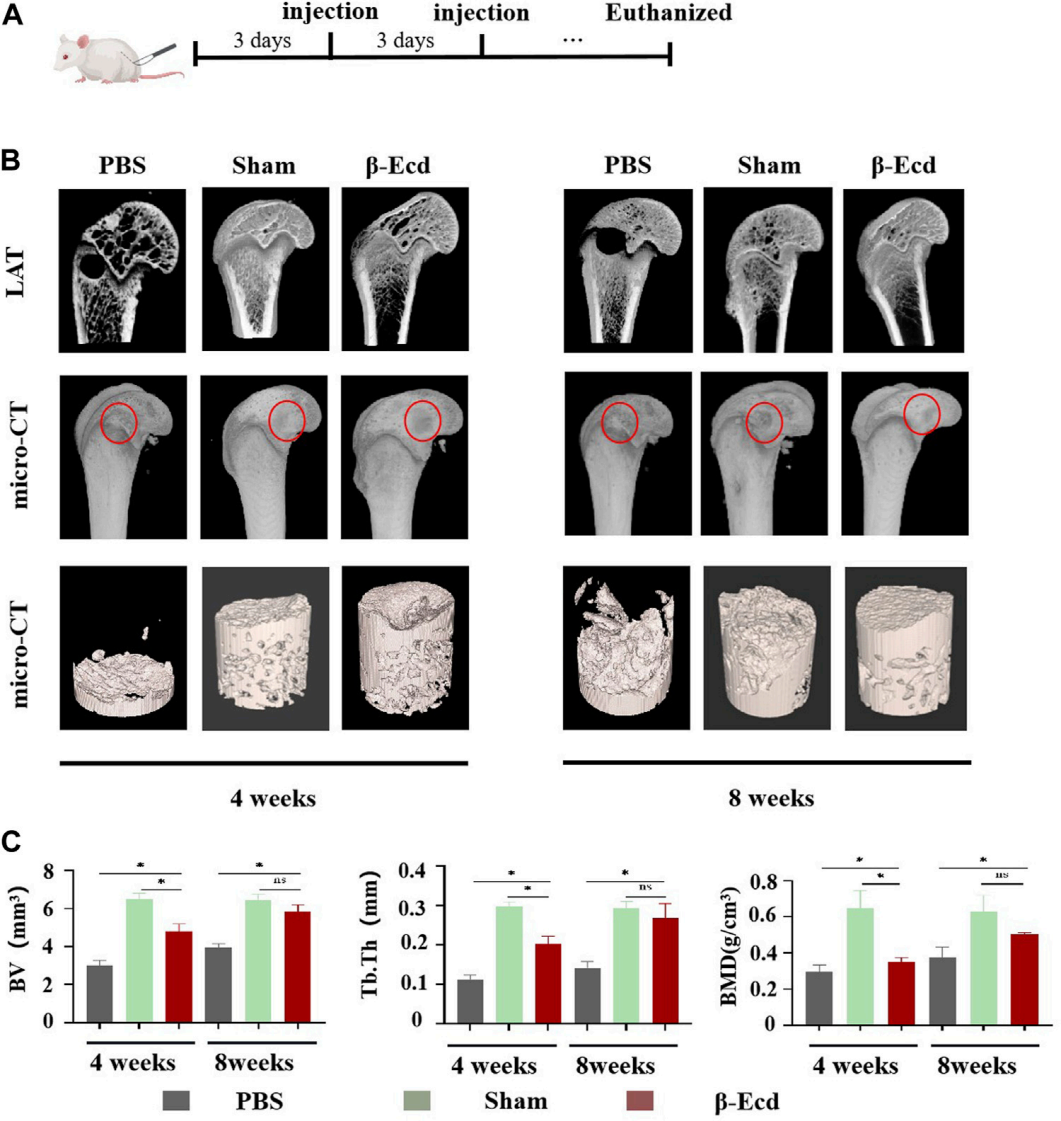
The results of the Micro-CT test show that β-Ecd accelerates bone healing in rats. **(A)** Critical size bone defect model of the distal femur and β-Ecd injection; **(B)** Micro-CT analysis of new bone formation, CT-vox software to identify new bone and analyze the distribution of new bone at 8 weeks. It can be seen that the new bone formation in the sham operation group and the β-Ecd group was significantly increased compared to the PBS group at 4 and 8 weeks, but there were no significant differences between the sham operation group and the β-Ecd group; **(C)** Statistics of new bone microstructural parameters in the 4 week and eighth week, including bone mineral density (BMD), bone tissue volume (BV), trabecular bone thickness (Tb.Th). Each group contained three replicates, and the data were analyzed by one-way ANOVA for multiple comparisons. *p < 0.05, ns, nosignificant.

**FIGURE 7:**
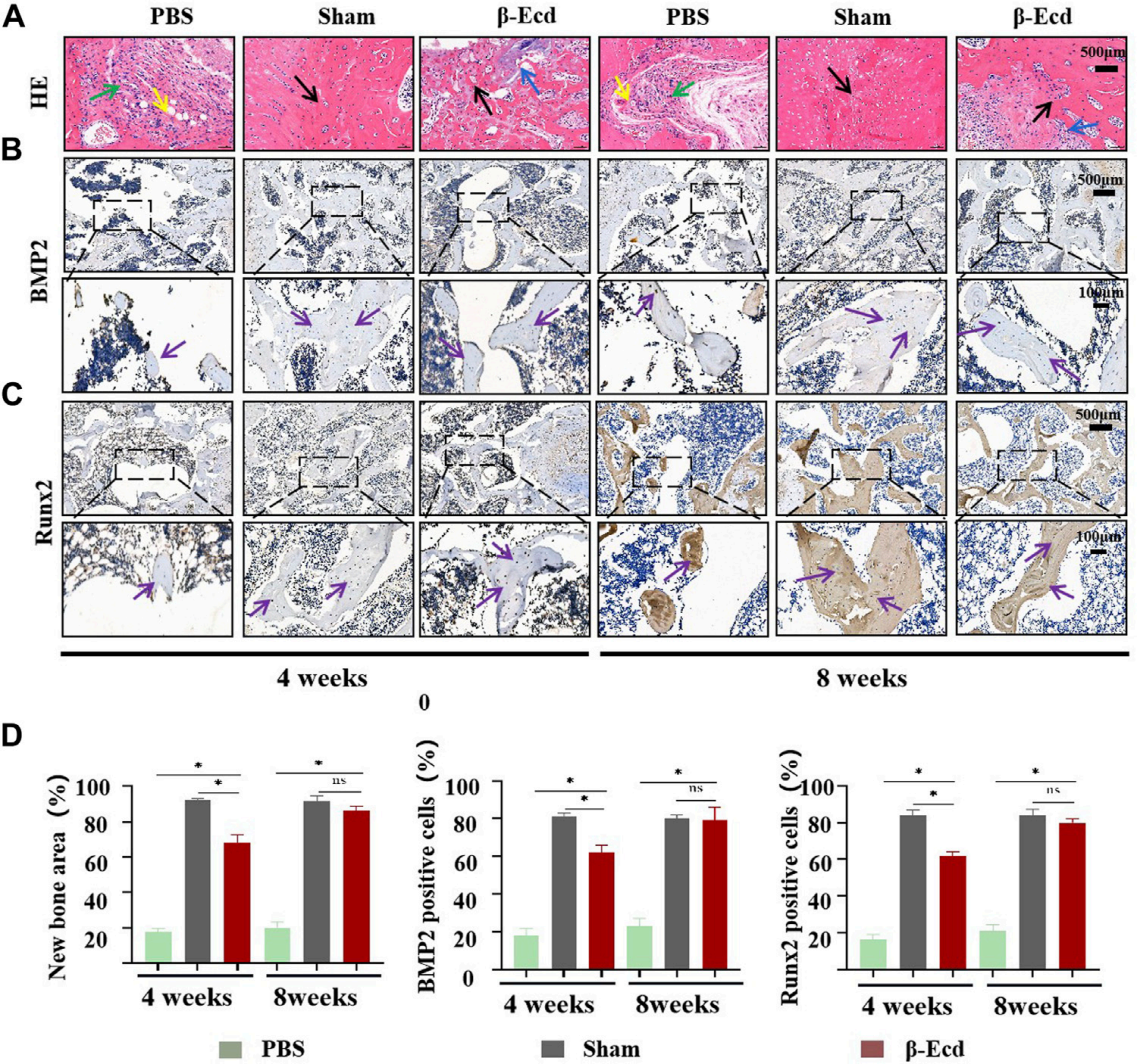
Histomorphological analysis of newly formed bone tissue in bone defect area and immunohistochemical evaluation of osteogenesis-related proteins in newly formed bone. **(A)** HE staining of bone tissue sections around the bone defect at 4 and 8 weeks; Green arrow: fibrous tissue; Yellow arrow: new capillary formation; Black arrow: bone tissue. **(B,C)** immunohistochemical detection of the expression of the BMP2/Runx2 protein in the new tissue in the area of the bone defect, the enlarged images in the black box are bone trabeculae or osteocytes; **(D)** The area of new bone tissue and the expression of the BMP2 protein in the three groups were statistically analyzed using ImageJ software. At 4 weeks, the expression of the BMP2/Runx2 protein was significantly increased in the bone defect area; At 8 weeks, the expression of the BMP2/Runx2 protein was significantly increased in the bone defect area, but there was no significant difference between the β-Ecd group and the sham-operated group. Each group contained three replicates, and the data were analyzed by one-way ANOVA for multiple comparisons. Purple arrow: BMP2/Runx2 protein positive site. *p < 0.05, ns, not significant.

As shown in Figure 6, micro-CT was used to evaluate changes in the femoral condylar defect in rats. On micro-CT reconstruction analysis, the images clearly showed characteristics of changes in the bone regeneration process. Compared to the control/PBS group, more new bone tissue had regenerated in the experimental group (Figure 6). On quantitative analysis, the volume and density of new bone tissue increased in the experimental group at 4 and 8 weeks (*p* < 0.05); however, there was no significant difference between the sham and experimental groups (*p* > 0.05) at 8 weeks. In addition to micro-CT, HE staining was used to detect histological changes in newly formed bone tissue. Unlike in the control group, the calcium phosphorus crystals in the bone tissue of the experimental group were arranged in a regular shape and the collagenous fibers were arranged in a circular shape. The direction of arrangement of the collagenous fibers was consistent with that of the bone cavities; this is a typical histomorphological characteristic of newly formed bone. Immunohistochemistry was used to detect the protein expression levels of BMP2, Smad4, Runx2, and Osterix. Compared to the control group, the expression of target protein in the experimental group was significantly increased. In addition, there was no significant difference in expression between the sham and control groups at 4 and 8 weeks (Figure 7).

## Discussion

Fractures, traumatic bone defects, and osteoporosis are increasingly prominent and common diseases worldwide, and adequate bone regeneration is the key their successful treatment. To our knowledge, bone regeneration is a common process of intramembranous ossification and endochondral ossification, initiated by periosteal bone progenitor cells, which first form avascular cartilage tissue and is then replaced by bone tissue (Slade and Chou 1998). BMSCs play an essential role in bone repair (Wang et al., 2013). Among the cytokines involved in bone formation, the transcription factor BMP2 is the most studied, but its induction mechanism in bone progenitor cells is poorly understood. This study showed that β-ecdysterone promotes bone formation and improves cell proliferation and differentiation by activating the BMP2/Smad/Runx2/Osterix signaling pathway, suggesting that β-ecdysterone can effectively improve bone volume and quality. Mechanistically, BMP2 binds to the BMPR-II receptor on the cell membrane and activates the BMPR-II receptor and BMPR-I receptor, regulates the binding of the downstream transcription factor Smad1/5/8 to the transcription factor Smad4, which is transferred to the nucleus and activates the downstream nuclear transcription factor Runx2. Runx2 further enhances Osterix fragment transcription and translation in the nucleic acid chain, promoting osteogenic proteins, extracellular matrix deposition, and calcium mineralization, leading to fracture regeneration. In general, our study suggests that β-ecdysterone is a positive regulator of bone regeneration, promoting BMSC proliferation and osteogenic differentiation.

β-ecdysterone has been shown to effectively protect mouse osteoblasts from glucocorticoid-induced apoptosis and autophagy (Tang et al., 2018a). It also blocks IL-1β-induced chondrocyte apoptosis and the inflammatory response by inhibiting NF-кB signaling (Zhang et al., 2014). In their *in vitro* study, Jian et al. (2013) (Xu et al., 2009) demonstrated that β-ecdysterone can induce BMP-2 dependent osteogenic differentiation and proliferation of human periodontal ligament stem cells through the extracellular signal-regulated kinase pathway. However, the results only evaluated the toxicity of β-ecdysterone and did not determine whether it promoted osteogenic differentiation of BMSCs. Data regarding the connection between estrogen receptors and BMP signaling are lacking. Studies have shown that cytokine sensitivity screening considers the BMP4 signaling pathway to be crucial for treating ER + breast cancer (Shee et al., 2019). In addition, dehydrodiconiferyl alcohol has been found to promote BMP-2-induced osteoblastogenesis through its agonistic effects on estrogen receptors (Lee et al., 2018). In their study, Pang et al. found that quercetin stimulates BMSC differentiation through an estrogen receptor-mediated pathway (Pang et al., 2018). We therefore suspect the presence of an interaction between BMP signaling and estrogen receptors; β-ecdysterone may have promoted osteogenic differentiation of BMSCs in our study by activating estrogen receptors *in vivo*.

Considering the importance of BMSCs in bone repair and reconstruction, we investigated the effects of β-ecdysterone on BMSCs *in vitro* and *in vivo*. *In vitro*, we used the MC3T3-E1 cell line, which includes osteogenic precursor cells cloned from the skull of C57BL/6 mice, to replace BMSC cells. Repeated subculture of this cell line has been reported to maintain the phenotype of high ALP activity of osteoblasts; it also promotes differentiation into osteoblasts and osteocytes *in vitro*, forming calcified bone tissue and mineral deposits of hydroxyapatite (Kunimatsu et al., 2018). This cell line has proven to be a viable model for exploring osteoblast proliferation, maturation, and differentiation (Gal et al., 2000) and is commonly used to study the effects of drugs on osteoblasts.

*In vitro* cell proliferation experiments showed that β-ecdysterone promoted MC3T3-E1 cell proliferation at 25–200 mM. In contrast, MC3T3-E1 cell proliferation and metabolism were inhibited after 7 days of culture or when the concentration was greater than 400 mM. Microscopically, cells reached fusion after 7 days of culture; this may explain why the effect of β-ecdysterone on the proliferation of MC3T3-E1 cells was limited to the early stage of cell culture. Our results also indicate that higher levels of β-ecdysterone exert a specific toxic effect on MC3T3-E1 cells. *In vivo* experiments were performed using 0 and 150 μM of β-ecdysterone (72 mg/kg), which was injected intraperitoneally in femoral condylar defect model rats. The results showed that the bone defect regenerated to different degrees after 8 weeks and the effect was most significant at 72 mg/kg. This result demonstrates that β-ecdysterone can promote osteoblast proliferation *in vivo* and has obvious biosafety. In previous in-vivo and in-vitro studies, the effect of β-ecdysterone on cell proliferation was complex (Tang et al., 2018b). In our study, β-ecdysterone at an appropriate concentration showed the ability to promote bone progenitor cell proliferation, which is essential for bone tissue regeneration; this is because the body needs enough bone cells to rebuild after fracture, and the number of cells that can be transplanted by autologous or allograft is limited.

Bone formation is a complex process. In addition to cell proliferation, deposition and mineralization of the extracellular matrix are also important (Jia et al., 2003). Therefore, ideal methods for bone regeneration must promote bone progenitor cell proliferation and stimulate osteogenic differentiation. In this study, MC3T3-E1 cells were cultured with different concentrations of β-ecdysterone to further verify the effect of β-ecdysterone on osteoprogenitor cell differentiation. The final data showed that β-ecdysterone significantly increased ALP activity in MC3T3-E1 cells, and the increase was more pronounced at a concentration of 200 mM Runx2 is an early phenotypic marker of mature osteoblasts similar to ALP, while COL-1 and OPN are late phenotypic markers of osteoblast differentiation (Zhang et al., 2010; Zhou et al., 2014). We demonstrated that β-ecdysterone induced significantly higher expression of the COL-1, OPN, and Runx2 gene or protein in MC3T3-E1 cells than in nonstimulated controls. These results suggest that β-ecdysterone stimulates early and late differentiation of bone progenitor cells.

For the in-vivo experiments, we selected a rat partial femoral condyle defect model. In the partial defect model, a defect is usually drilled into the side of the bone to create an area of injury. Drilling through the cortical bone may extend to the underlying cancellous bone or marrow cavity. In this model, only one bone is usually injured; notably, certain cortical bone defects are simple to operate and can simulate the steps of stable fracture healing (McGovern et al., 2018). They offer many advantages over other closed and open fractures, including reduced morbidity in animals and better histomorphometric analysis. Micro-CT values and immunohistochemical staining results of the rat bone defect model showed greater new bone formation (based on mineralization measurement) in the group treated with β-ecdysterone than in the control/PBS group. In conjunction, these results suggest that β-ecdysterone stimulates osteogenic differentiation of bone progenitors at different stages *in vitro* and *in vivo*.

BMPs play a vital role in the osteogenic differentiation of different cell lines (Urist and Strates 2009; Long 2011). Through gene sequencing and differential expression analysis of osteogenic related genes in different treatment groups of MC3T3-E1 cells, we found that the genes detected in different groups of cells had significant differences in signal pathway enrichment and cell function. The BMP signaling pathway genes were ranked among the top 20 and the differences were statistically significant. BMP-2 has been reported to be a crucial regulatory factor in the BMP pathway, which can enhance the osteogenic differentiation of human BMSCs (Peng et al., 2003). Therefore, our analysis of the results of gene sequencing suggested that β-ecdysterone-enhanced osteogenic differentiation of bone progenitor cells was closely related to the BMP2 signaling pathway. Similarly, we found that β-ecdysterone upregulates BMP-2 expression at mRNA and protein levels, and the BMP-2 signaling pathway inhibitor noggin can counteract this effect *in vitro*. Although noggin has been observed to be nonspecific for BMP-2 (Secondini et al., 2011), genetic tests showed that β-ecdysterone did not significantly increase BMP signaling in MC3T3-E1 cells. These results suggest that the BMP-2 signaling pathway plays an essential role in β-ecdysterone-induced osteogenic differentiation of bone progenitor cells.

Regarding the induction mechanism of BMP-2, many studies have shown that it is related to the MAPK signaling pathway (Park et al., 2019). Previous studies have reported that the ERK pathway is involved in the differentiation of periodontal ligament cells and osteoblasts (Tóth et al., 2008), but our results suggest that the increased expression of BMP-2 and other osteogenic proteins and genes is directly related to the BMP2/Smad/Runx2/Osterix pathway. The noggin inhibitor can abrogate this effect. According to our data and previous studies (Qiao et al., 2005; Wang et al., 2012; Fischerauer et al., 2013), we speculate that the mechanism of action involves BMP-2 active the BMP type 2 receptor, but it interacts and activates the BMP type 1 receptor which then the BMP type 1 receptor activates downstream signaling pathways, by phosphorylation of Smad1, Smad5, or Smad8. Smad1/5/8 activates and binds to Smad4 and enters the cell nucleus to regulate the transcription function of specific genes. The Smad protein, as a coregulatory, interacts with Runx2 to participate in osteoblast phenotypic gene expression and differentiation (Phimphilai et al., 2006). In addition, Runx2 can interact with osteoblast specifics acting element 2 in the osteocalcin promoter region to stimulate osteocalcin expression. There are osteoblast specifics acting element 2-like elements in the promoter regions of osteoblast-related genes such as type I collagen, osteocalcin, and osteopontin, and Runx2 can bind to these osteoblast specifics acting element 2-like elements to activate gene expression (Liu and Lee 2013); however, this mechanism requires further study.

In summary, β-ecdysterone can be used as a safe and effective agent for bone regeneration to resolve insufficient bone regeneration and severe osteoporosis caused by decreased osteogenic capacity. Thus, β-ecdysterone has excellent research value and application prospects. Furthermore, it remains to be explored whether β-ecdysterone can be incorporated into bone regeneration biomaterials to promote bone tissue regeneration for the treatment of critical bone defects in the future.

## Conclusion

This study is the first to demonstrate that β-ecdysterone has good biosafety in mammals *in vitro* and *in vivo* and can promote proliferation and induce osteogenic differentiation of bone progenitors through the BMP2/Smad/Runx2/Osterix signaling pathway. This indicates its considerable potential as a therapeutic agent for bone regeneration and repair.

## Data Availability

The datasets presented in this study can be found in online repositories. The names of the repository/repositories and accession number(s) can be found in the article/Supplementary Material.

## References

[B1] Abdallah B. M. Haack-Sørensen M. Burns J. S. Elsnab B. Jakob F. Hokland P. (2005). Maintenance of Differentiation Potential of Human Bone Marrow Mesenchymal Stem Cells Immortalized by Human Telomerase Reverse Transcriptase Gene Despite of Extensive Proliferation. Biochem. biophysical Res. Commun. 326, 527–538. 10.1016/j.bbrc.2004.11.059 15596132

[B2] Abiramasundari G. Mohan Gowda C. M. Sreepriya M. (2018). Selective Estrogen Receptor Modulator and Prostimulatory Effects of Phytoestrogen β-ecdysone in Tinospora Cordifolia on Osteoblast Cells. J. Ayurveda Integr. Med. 9, 161–168. 10.1016/j.jaim.2017.04.003 30166229 PMC6148058

[B3] Cai H. Zou J. Wang W. Yang A. (2021). BMP2 Induces hMSC Osteogenesis and Matrix Remodeling. Mol. Med. Rep. 23, 11764. 10.3892/mmr.2020.11764 PMC7751477 33300084

[B4] Catalán R. E. Martinez A. M. Aragones M. D. Miguel B. G. Robles A. Godoy J. E. (1985). Alterations in Rat Lipid Metabolism Following Ecdysterone Treatment. Comp. Biochem. physiology. B, Comp. Biochem. 81, 771 10.1016/0305-0491(85)90403-1 4028688

[B5] Chen Z. Wang X. Shao Y. Shi D. Chen T. Cui D. (2011). Synthetic Osteogenic Growth Peptide Promotes Differentiation of Human Bone Marrow Mesenchymal Stem Cells to Osteoblasts via RhoA/ROCK Pathway. Mol. Cell Biochem. 358, 221–227. 10.1007/s11010-011-0938-7 21739156

[B6] Clark D. P. Badea C. T. (2021). Advances in Micro-CT Imaging of Small Animals. Phys. Medica 88, 175–192. 10.1016/j.ejmp.2021.07.005 PMC8447222 34284331

[B7] da Silva Meirelles L. Fontes A. M. Covas D. T. Caplan A. I. (2009). Mechanisms Involved in the Therapeutic Properties of Mesenchymal Stem Cells. Cytokine & growth factor Rev. 20, 419–427. 10.1016/j.cytogfr.2009.10.002 19926330

[B8] Dai W.-W. Wang L.-B. Jin G.-Q. Wu H.-J. Zhang J. Wang C.-L. (2017). Beta-Ecdysone Protects Mouse Osteoblasts from Glucocorticoid-Induced Apoptosis *In Vitro* . Planta Med. 83, 888–894. 10.1055/s-0043-107808 28388784

[B9] Feng C. Xiao L. Yu J. C. Li D. Y. Tang T. Y. Liao W. (2020). Simvastatin Promotes Osteogenic Differentiation of Mesenchymal Stem Cells in Rat Model of Osteoporosis through BMP-2/Smads Signaling Pathway. Eur. Rev. Med. Pharmacol. Sci. 24, 434–443. 10.26355/eurrev_202001_19943 31957858

[B10] Fischerauer E. E. Manninger M. Seles M. Janezic G. Pichler K. Ebner B. (2013).BMP-6 and BMPR-1a Are Up-Regulated in the Growth Plate of the Fractured Tibia. J. Orthop. Res., 31, 357–363. 10.1002/jor.22238 23097200

[B11] Gal T. J. Munoz-Antonia T. Muro-Cacho C. A. Klotch D. W. (2000). Radiation Effects on Osteoblasts *In Vitro*: a Potential Role in Osteoradionecrosis. Arch. Otolaryngol. Head. Neck Surg. 126, 1124–1128. 10.1001/archotol.126.9.1124 10979127

[B12] Gao L. Cai G. Shi X. (2008). BETA.-Ecdysterone Induces Osteogenic Differentiation in Mouse Mesenchymal Stem Cells and Relieves Osteoporosis. Biol. Pharm. Bull. 31, 2245–2249. 10.1248/bpb.31.2245 19043207

[B13] Hak D. J. Fitzpatrick D. Bishop J. A. Marsh J. L. Tilp S. Schnettler R. (2014). Delayed Union and Nonunions: Epidemiology, Clinical Issues, and Financial Aspects. Injury 45 (Suppl. 2), S3–S7. 10.1016/j.injury.2014.04.002 24857025

[B14] Jia T.-L. Wang H.-Z. Xie L.-P. Wang X.-Y. Zhang R.-Q. (2003). Daidzein Enhances Osteoblast Growth that May Be Mediated by Increased Bone Morphogenetic Protein (BMP) Production. Biochem. Pharmacol. 65, 709–715. 10.1016/s0006-2952(02)01585-x 12628484

[B15] Jian C.-X. Liu X.-F. Hu J. Li C.-J. Zhang G. Li Y. (2013). 20-Hydroxyecdysone-induced Bone Morphogenetic Protein-2-dependent Osteogenic Differentiation through the ERK Pathway in Human Periodontal Ligament Stem Cells. Eur. J. Pharmacol. 698, 48–56. 10.1016/j.ejphar.2012.07.044 23397605

[B16] Ko F. C. Sumner D. R. (2021). How Faithfully Does Intramembranous Bone Regeneration Recapitulate Embryonic Skeletal Development? Dev. Dyn. 250, 377–392. 10.1002/dvdy.240 32813296

[B17] Kunimatsu R. Gunji H. Tsuka Y. Yoshimi Y. Awada T. Sumi K. (2018). Effects of High-Frequency Near-Infrared Diode Laser Irradiation on the Proliferation and Migration of Mouse Calvarial Osteoblasts. Lasers Med. Sci. 33, 959–966. 10.1007/s10103-017-2426-0 29302842

[B18] Lee W. Ko K. R. Kim H.-K. Lim S. Kim S. (2018). Dehydrodiconiferyl Alcohol Promotes BMP-2-Induced Osteoblastogenesis through its Agonistic Effects on Estrogen Receptor. Biochem. biophysical Res. Commun. 495, 2242–2248. 10.1016/j.bbrc.2017.12.079 29253565

[B19] Li Z. Helms J. A. (2021). Drill Hole Models to Investigate Bone Repair. Methods Mol. Biol. Clift. N.J.) 2221, 193–204. 10.1007/978-1-0716-0989-7_12 32979205

[B20] Lin Z.-Y. Duan Z.-X. Guo X.-D. Li J.-F. Lu H.-W. Zheng Q.-X. (2010). Bone Induction by Biomimetic PLGA-(PEG-ASP)n Copolymer Loaded with a Novel Synthetic BMP-2-Related Peptide *In Vitro* and *In Vivo* . J. Control. Release 144, 190–195. 10.1016/j.jconrel.2010.02.016 20184932

[B21] Liu Q. Zhang X. Jiao Y. Liu X. Wang Y. Li S. L. (2018). *In Vitro* cell Behaviors of Bone Mesenchymal Stem Cells Derived from Normal and Postmenopausal Osteoporotic Rats. Int. J. Mol. Med. 41, 669–678. 10.3892/ijmm.2017.3280 29207050 PMC5752170

[B22] Liu T. M. Lee E. H. (2013). Transcriptional Regulatory Cascades in Runx2-dependent Bone Development. Tissue Eng. Part B Rev. 19, 254–263. 10.1089/ten.teb.2012.0527 23150948 PMC3627420

[B23] Long F. (2011). Building Strong Bones: Molecular Regulation of the Osteoblast Lineage. Nat. Rev. Mol. Cell Biol. 13, 27–38. 10.1038/nrm3254 22189423

[B24] McGovern J. A. Griffin M. Hutmacher D. W. (2018). Animal Models for Bone Tissue Engineering and Modelling Disease. Dis. Model. Mech. 11. 10.1242/dmm.033084 PMC5963860 29685995

[B25] Miyazono K. Kamiya Y. Morikawa M. (2010). Bone Morphogenetic Protein Receptors and Signal Transduction. J. Biochem. 147, 35–51. 10.1093/jb/mvp148 19762341

[B26] Pang X. G. Cong Y. Bao N. R. Li Y. G. Zhao J. N. (2018). Quercetin Stimulates Bone Marrow Mesenchymal Stem Cell Differentiation through an Estrogen Receptor-Mediated Pathway. Biomed. Res. Int. 2018, 4178021. 10.1155/2018/4178021 29736392 PMC5875037

[B27] Park M. Choi H. K. An J. H. (2019). Taurine Activates BMP-2/Wnt3a-Mediated Osteoblast Differentiation and Mineralization via Akt and MAPK Signaling. Iran. J. Public Health 48, 1960 31970094 PMC6961198

[B28] Peng Y. Kang Q. Cheng H. Li X. Sun M. H. Jiang W. (2003). Transcriptional Characterization of Bone Morphogenetic Proteins (BMPs)-Mediated Osteogenic Signaling. J. Cell. Biochem. 90, 1149–1165. 10.1002/jcb.10744 14635189

[B29] Percival C. J. Richtsmeier J. T. (2013). Angiogenesis and Intramembranous Osteogenesis. Dev. Dyn. 242, 909–922. 10.1002/dvdy.23992 23737393 PMC3803110

[B30] Phillips A. M. (2005). Overview of the Fracture Healing Cascade. Injury 36 Suppl 3 (Suppl. 3), S5–S7. 10.1016/j.injury.2005.07.027 16188551

[B31] Phimphilai M. Zhao Z. Boules H. Roca H. Franceschi R. T. (2006). BMP Signaling Is Required for RUNX2-dependent Induction of the Osteoblast Phenotype. J. Bone Min. Res. 21, 637–646. 10.1359/jbmr.060109 PMC2435171 16598384

[B32] Qiao B. Padilla S. R. Benya P. D. (2005). Transforming Growth Factor (TGF)-β-activated Kinase 1 Mimics and Mediates TGF-β-Induced Stimulation of Type II Collagen Synthesis in Chondrocytes Independent of Col2a1 Transcription and Smad3 Signaling. J. Biol. Chem. 280, 17562–17571. 10.1074/jbc.m500646200 15743758

[B33] Scarfì S. (2016). Use of Bone Morphogenetic Proteins in Mesenchymal Stem Cell Stimulation of Cartilage and Bone Repair. World J. Stem Cells 8, 1–12. 10.4252/wjsc.v8.i1.1 26839636 PMC4723717

[B34] Schindeler A. McDonald M. M. Bokko P. Little D. G. (2008). Bone Remodeling during Fracture Repair: The Cellular Picture. Seminars Cell & Dev. Biol. 19, 459–466. 10.1016/j.semcdb.2008.07.004 18692584

[B35] Schubert H. Eiselt M. Walter B. Fritz H. Brodhun M. Bauer R. (2012). Isoflurane/nitrous Oxide Anesthesia and Stress-Induced Procedures Enhance Neuroapoptosis in Intrauterine Growth-Restricted Piglets. Intensive Care Med. 38, 1205–1214. 10.1007/s00134-012-2576-2 22576279

[B36] Secondini C. Wetterwald A. Schwaninger R. Thalmann G. N. Cecchini M. G. (2011). The Role of the BMP Signaling Antagonist Noggin in the Development of Prostate Cancer Osteolytic Bone Metastasis. PloS one 6, e16078. 10.1371/journal.pone.0016078 21249149 PMC3020964

[B37] Shee K. Jiang A. Varn F. S. Liu S. Traphagen N. A. Owens P. (2019). Cytokine Sensitivity Screening Highlights BMP4 Pathway Signaling as a Therapeutic Opportunity in ER + Breast Cancer. FASEB J. 33, 1644–1657. 10.1096/fj.201801241r 30161001 PMC6338642

[B38] Slade J. F. Chou K. H. (1998). Bony Tissue Repair. J. Hand Ther. 11, 118–124. 10.1016/s0894-1130(98)80008-2 9602968

[B39] Smagghe G. Vanhassel W. Moeremans C. De Wilde D. Goto S. Loeb M. J. (2005). Stimulation of Midgut Stem Cell Proliferation and Differentiation by Insect Hormones and Peptides. Ann. N. Y. Acad. Sci. 1040, 472–475. 10.1196/annals.1327.094 15891093

[B40] Takigawa M. (2013). CCN2: a Master Regulator of the Genesis of Bone and Cartilage. J. Cell Commun. Signal. 7, 191–201. 10.1007/s12079-013-0204-8 23794334 PMC3709051

[B41] Tang Y. H. Yue Z. S. Li G. S. Zeng L. R. Xin D. W. Hu Z. Q. (2018a). Effect of βecdysterone on Glucocorticoidinduced Apoptosis and Autophagy in Osteoblasts. Mol. Med. Rep. 17, 158–164. 10.3892/mmr.2017.7840 29115419 PMC5780097

[B42] Tang Y. H. Yue Z. S. Xin D. W. Zeng L. R. Xiong Z. F. Hu Z. Q. (2018b). βEcdysterone Promotes Autophagy and Inhibits Apoptosis in Oteoporotic Rats. Mol. Med. Rep. 17, 1591–1598. 10.3892/mmr.2017.8053 29138818 PMC5780099

[B43] Tóth N. Szabó A. Kacsala P. Héger J. Zádor E. (2008). 20-Hydroxyecdysone Increases Fiber Size in a Muscle-specific Fashion in Rat. Phytomedicine 15, 691–698. 10.1016/j.phymed.2008.04.015 18585021

[B44] Toth Z. Ward A. Tang S. Y. McBride-Gagyi S. (2021). Sexual Differences in Bone Porosity, Osteocyte Density, and Extracellular Matrix Organization Due to Osteoblastic-specific Bmp2 Deficiency in Mice. Bone 150, 116002. 10.1016/j.bone.2021.116002 33971313 PMC8217247

[B45] Urist M. R. Strates B. S. (2009). The Classic: Bone Morphogenetic Protein. Clin. Orthop. Relat. Res. 467, 3051–3062. 10.1007/s11999-009-1068-3 19727989 PMC2772914

[B46] Wang X. Wang Y. Gou W. Lu Q. Peng J. Lu S. (2013). Role of Mesenchymal Stem Cells in Bone Regeneration and Fracture Repair: a Review. Int. Orthop. (SICOT) 37, 2491–2498. 10.1007/s00264-013-2059-2 PMC3843208 23948983

[B47] Wang Y. Li L. Zheng Y. Yuan G. Yang G. He F. (2012). BMP Activity Is Required for Tooth Development from the Lamina to Bud Stage. J. Dent. Res. 91, 690–695. 10.1177/0022034512448660 22592126 PMC3383849

[B48] Wen F. Yu J. He C. J. Zhang Z. W. Yang A. F. (2019). Βecdysterone Protects against Apoptosis by Promoting Autophagy in Nucleus Pulposus Cells and Ameliorates Disc Degeneration. Mol. Med. Rep. 19, 2440–2448. 10.3892/mmr.2019.9861 30664184

[B49] Xu J. Wang W. Kapila Y. Lotz J. Kapila S. (2009). Multiple Differentiation Capacity of STRO-1+/CD146^+^PDL Mesenchymal Progenitor Cells. Stem cells Dev. 18, 487–496. 10.1089/scd.2008.0113 18593336 PMC2702120

[B50] Yan Y. Chen H. Zhang H. Guo C. Yang K. Chen K. (2019). Vascularized 3D Printed Scaffolds for Promoting Bone Regeneration. Biomaterials 190, 197. 10.1016/j.biomaterials.2018.10.033 30415019

[B58] Yang C. Liu X. Zhao K. Zhu Y. Hu B. Zhou Y. (2019). miRNA-21 Promotes Osteogenesis via the PTEN/PI3K/Akt/HIF-1α Pathway and Enhances Bone Regeneration in Critical Size Defects. Stem Cell Res Ther. 10, 65. 10.1186/s13287-019-1168-2 30795815 PMC6387542

[B51] Ying X. Cheng S. Wang W. Lin Z. Chen Q. Zhang W. (2011). Effect of Boron on Osteogenic Differentiation of Human Bone Marrow Stromal Cells. Biol. Trace Elem. Res. 144, 306–315. 10.1007/s12011-011-9094-x 21625915

[B52] Yoshida T. Otaka T. Uchiyama M. Ogawa S. (1971). Effect of Ecdysterone on Hyperglycemia in Experimental Animals. Biochem. Pharmacol. 20, 3263–3268. 10.1016/0006-2952(71)90431-x 5132876

[B53] Yu B. Huo L. Liu Y. Deng P. Szymanski J. Li J. (2018). PGC-1α Controls Skeletal Stem Cell Fate and Bone-Fat Balance in Osteoporosis and Skeletal Aging by Inducing TAZ. Cell stem Cell 23, 615–623. 10.1016/j.stem.2018.09.001 30244868 PMC6613582

[B54] Zhang L. Hu Y. Sun C.-y. Li J. Guo T. Huang J. (2010). Lentiviral shRNA Silencing of BDNF Inhibitsin Vivomultiple Myeloma Growth and Angiogenesis via Down-Regulated Stroma-Derived VEGF Expression in the Bone Marrow Milieu. Cancer Sci. 101, 1117–1124. 10.1111/j.1349-7006.2010.01515.x 20331634 PMC11158522

[B55] Zhang X. Xu X. Xu T. Qin S. (2014). β-Ecdysterone Suppresses Interleukin-1β-Induced Apoptosis and Inflammation in Rat Chondrocytes via Inhibition of NF-Κb Signaling Pathway. Drug Dev. Res. 75, 195–201. 10.1002/ddr.21170 24648308

[B59] Zhao S. J. Kong F. Q. Jie J. Li Q. Liu H. Xu A. D. (2020). Macrophage MSR1 Promotes BMSC Osteogenic Differentiation and M2-Like Polarization by Activating PI3K/AKT/GSK3β/β-Catenin Pathway. Theranostics 10, 17–35. 10.7150/thno.36930 31903103 PMC6929615

[B56] Zhou P. R. Liu H. J. Liao E. Y. Zhang Z. L. Chen D. C. Liu J. (2014). LRP5 Polymorphisms and Response to Alendronate Treatment in Chinese Postmenopausal Women with Osteoporosis. Pharmacogenomics 15, 821–831. 10.2217/pgs.14.12 24897288

[B57] Zou Y. Wang R. Guo H. Dong M. (2015). Phytoestrogen β-Ecdysterone Protects PC12 Cells against MPP+-Induced NeurotoxicityIn Vitro: Involvement of PI3K-Nrf2-Regulated Pathway. Toxicol. Sci. 147, 28–38. 10.1093/toxsci/kfv111 26048653

