# Supplementary material for: β-Ecdysterone Enhanced Bone Regeneration Through the BMP-2/SMAD/RUNX2/Osterix Signaling Pathway
Source: Front Cell Dev Biol. 2022 May 20;10:883228. doi: 10.3389/fcell.2022.883228 (PMC9164109; doi:10.3389/fcell.2022.883228)
Supplement: Supplementary file 1 [file DataSheet1.docx]

**
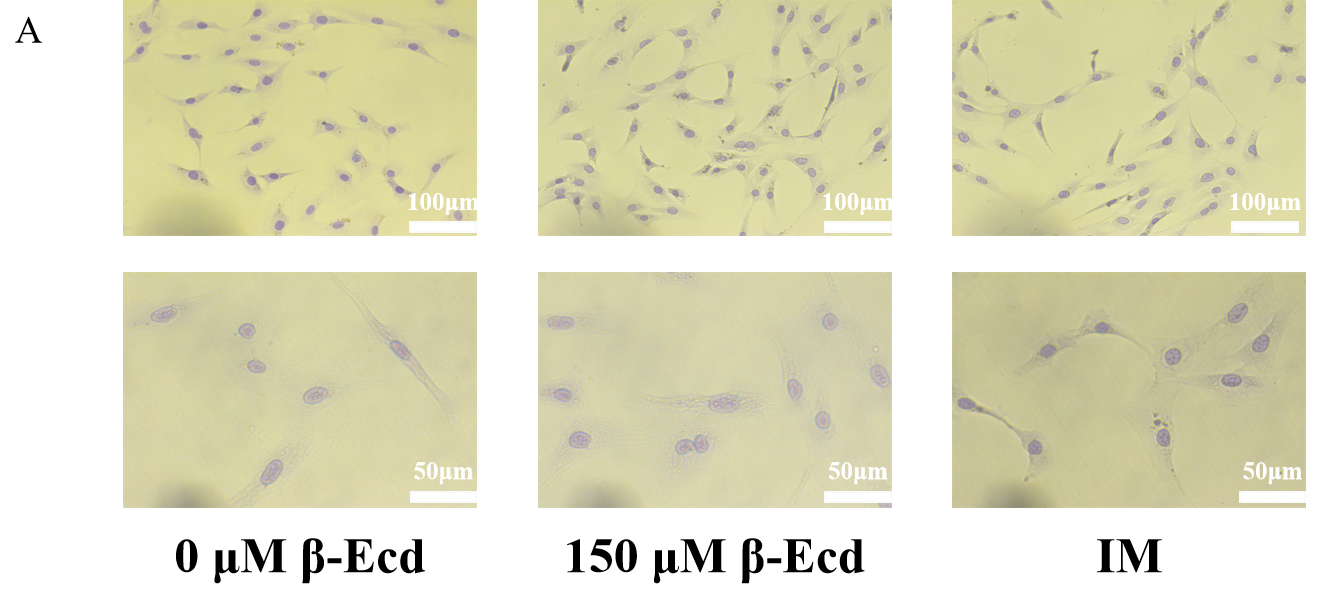
**

Note**:** This figure shows the antibody controls in our study (OPN). OPN primary antibody was not added to the MC3T3-E1 cells, only secondary antibody and hematoxylin were added. The nucleus was observed in the cells, and no OPN expression was observed in the cytoplasm.


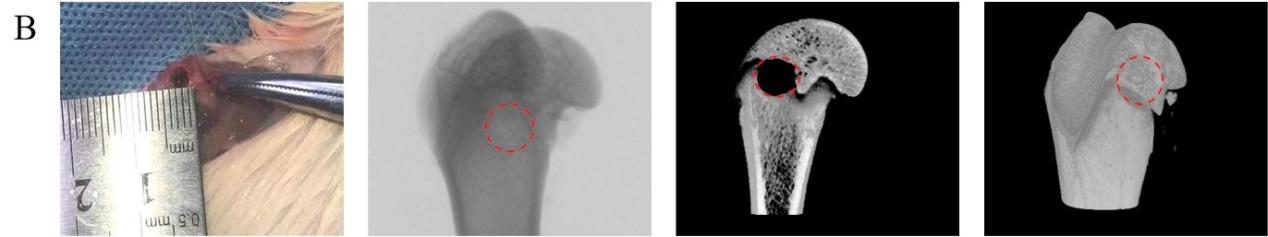


Note：The rats model made and the specimen was tested.

The datasets RNA sequence analysis and gene enrichment analysis for this study can be found in the: https://www.ncbi.nlm.nih.gov/geo/query/acc.cgi?acc=GSE198010.
